# Darunavir/cobicistat/emtricitabine/tenofovir alafenamide in treatment-experienced, virologically suppressed patients with HIV-1: subgroup analyses of the phase 3 EMERALD study

**DOI:** 10.1186/s12981-019-0235-1

**Published:** 2019-08-29

**Authors:** Gregory D. Huhn, Joseph J. Eron, Pierre-Marie Girard, Chloe Orkin, Jean-Michel Molina, Edwin DeJesus, Romana Petrovic, Donghan Luo, Erika Van Landuyt, Erkki Lathouwers, Richard E. Nettles, Kimberley Brown, Eric Y. Wong

**Affiliations:** 1 The Ruth M. Rothstein CORE Center, 2020 W Harrison Street, Chicago, IL 60612 USA; 2 University of North Carolina School of Medicine, 321 S Columbia St., Chapel Hill, NC 27516 USA; 3 Hôpital St Antoine, Assistance Publique-Hôpitaux de Paris and Sorbonne University, 184 rue du Faubourg Saint-Antoine, 75012 Paris, France; 4 Queen Mary University of London, Mile End Rd, Bethnal Green, London, E1 4NS UK; 5 University of Paris Diderot, St-Louis Hospital, 12 Rue de la Grange aux Belles, 75010 Paris, France; 6 Orlando Immunology Center, 1707 North Mills Avenue, Orlando, FL 32803 USA; 7 Janssen Research & Development, Turnhoutseweg 30, 2340, Beerse, Belgium; 8 Janssen Research & Development, LLC, 1125 Trenton-Harbourton Road, Titusville, NJ 08560 USA; 9 Janssen Scientific Affairs, LLC, 1125 Trenton-Harbourton Road, Titusville, NJ 08560 USA

**Keywords:** HIV-1, Darunavir, Antiretroviral, Protease inhibitor, Single-tablet regimen, Switch study

## Abstract

**Background:**

Darunavir/cobicistat/emtricitabine/tenofovir alafenamide (D/C/F/TAF) 800/150/200/10 mg is a once-daily, single-tablet regimen for treatment of HIV-1 infection. The efficacy/safety of switching to D/C/F/TAF versus continuing boosted protease inhibitor (bPI) + emtricitabine/tenofovir disoproxil fumarate (control) were demonstrated in a phase 3, randomized study (EMERALD) of treatment-experienced, virologically suppressed adults through week 48. The objective of this analysis was to evaluate EMERALD outcomes across subgroups of patients based on demographic characteristics, prior treatment experience, and baseline antiretroviral regimen.

**Methods:**

EMERALD patients were virologically suppressed (viral load [VL] < 50 copies/mL for ≥ 2 months at screening). Prior non-darunavir virologic failure (VF) was allowed. Primary endpoint was proportion of patients with virologic rebound (confirmed VL ≥ 50 copies/mL) cumulative through week 48. Virologic response was VL < 50 copies/mL (FDA snapshot). Safety was assessed by adverse events, renal proteinuria markers, and bone mineral density. Outcomes were examined for prespecified subgroups by age (≤/> 50 years), gender, race (black/non-black), prior number of antiretrovirals used (4/5/6/7/> 7), prior VF (0/≥ 1), baseline bPI (darunavir/atazanavir or lopinavir), and baseline boosting agent (ritonavir/cobicistat).

**Results:**

Among 1141 patients in the D/C/F/TAF (n = 763) and control (n = 378) arms, virologic rebound rates (2.5% and 2.1%, respectively) were similar, and this was consistent across all subgroups. Virologic response rates ranged from 91 to 97% (D/C/F/TAF) and 89 to 99% (control) across all subgroups, with differences between treatment arms of 0 and 6%. Adverse event rates were low in both arms and across subgroups. Improvements in renal and bone parameters were observed with D/C/F/TAF across demographic subgroups.

**Conclusions:**

For treatment-experienced, virologically suppressed patients, switching to D/C/F/TAF was highly effective and safe, regardless of demographic characteristics, prior treatment experience, or pre-switch bPI.

*Trial registration* ClinicalTrials.gov Identifier: NCT02269917. Registered 21 October 2014. https://clinicaltrials.gov/ct2/show/NCT02269917

**Electronic supplementary material:**

The online version of this article (10.1186/s12981-019-0235-1) contains supplementary material, which is available to authorized users.

## Background

Darunavir/cobicistat/emtricitabine/tenofovir alafenamide (D/C/F/TAF) 800/150/200/10 mg is an oral, once-daily (QD) single-tablet regimen (STR) for the treatment of human immunodeficiency virus (HIV)-1 infection. The darunavir component has demonstrated efficacy and safety, as well as a high barrier to resistance [1, 2]. A darunavir-based regimen is recommended in guidelines from the European AIDS Clinical Society, and the US Department of Health and Human Services recommends a darunavir-based regimen in clinical situations such as when there are adherence concerns or resistance data are not available prior to treatment initiation (i.e., when a high barrier to resistance is important) [3, 4].

In the phase 3 EMERALD study, treatment-experienced, virologically suppressed adults switched to D/C/F/TAF or remained on a boosted protease inhibitor (bPI) + emtricitabine/tenofovir disoproxil fumarate (TDF) regimen; D/C/F/TAF was noninferior versus the control regimen (4% margin) for cumulative virologic rebound through week 48 [5]. High virologic response rates and a favorable safety profile were also shown for D/C/F/TAF. Notably, EMERALD had less strict enrollment criteria for treatment experience compared with other recent switch studies, allowing a broader range of patients that may be more representative of patients who require a switch in clinical practice [6–8]. Specifically, patients with prior experience with multiple antiretrovirals (ARVs) and/or prior virologic failure (VF) and those with an HIV-1 RNA blip prior to screening were eligible to enroll, and there were no restrictions on emtricitabine or tenofovir resistance-associated mutations (RAMs). To examine the efficacy and safety of D/C/F/TAF in specific populations of patients who may switch to this regimen, we evaluated results from EMERALD across subgroups of patients based on demographic characteristics, prior treatment experience, and ARV regimen used at baseline.

## Methods

### Study design

EMERALD was a phase 3, randomized, active-controlled, multicenter, open-label trial evaluating efficacy, resistance development, and safety in treatment-experienced, virologically suppressed patients with HIV-1 who switched from a bPI + emtricitabine/TDF regimen to D/C/F/TAF, or continued on their current regimen. The bPI was darunavir or atazanavir (with ritonavir or cobicistat) or lopinavir (with ritonavir). Detailed study methods for EMERALD have previously been described [5].

### Analyses

Efficacy was assessed by the proportion of patients with cumulative virologic rebound (confirmed HIV-1 RNA ≥ 50 copies/mL) through week 48 (primary endpoint) and virologic response (HIV-1 RNA < 50 copies/mL) and VF at week 48 (US Food and Drug Administration [FDA] snapshot approach) [9]. The difference (95% confidence interval [CI]) between the D/C/F/TAF and control arms for virologic rebound and virologic response for subgroups was calculated using exact CIs. The trial was not powered for statistical testing of treatment-by-subgroup interaction.

To evaluate post-baseline resistance, genotyping was performed in patients with virologic rebound who also had a viral load measurement of ≥ 400 copies/mL at the time of VF, at later time points, or at discontinuation.

Safety was assessed by monitoring adverse events (AEs). For subgroups based on demographic characteristics, additional safety analyses are reported (renal and lipid laboratory parameters, and, for patients in the bone investigation substudy, changes in bone mineral density [BMD]).

Prespecified subgroup analyses were performed on all randomized patients who received ≥ 1 dose of study drug. Demographic subgroups were based on age (≤ 50 vs > 50 years), gender (men vs women), and race (non-black/African American vs black/African American). Prior treatment experience subgroups were based on the number of prior ARVs used (including those used at baseline; 4 vs 5 vs 6 vs 7 vs > 7 prior ARVs used) and prior VF (0 vs ≥ 1 prior VF). Subgroups corresponding to ARV regimen at baseline were based on bPI (darunavir [with ritonavir or cobicistat] vs atazanavir [with ritonavir or cobicistat] or lopinavir [with ritonavir]) and boosting agent (ritonavir [with darunavir, atazanavir, or lopinavir] vs cobicistat [with darunavir or atazanavir]).

Additionally, because polypharmacy may be a concern among older individuals, age subgroups (≤ 50 vs > 50 years) were further divided for assessment based on the presence or absence of polypharmacy (defined as ≥ 5 [non-ARV] concomitant medications at baseline).

## Results

### Study population

A total of 1141 patients were randomized and treated in EMERALD, including 763 in the D/C/F/TAF arm and 378 in the control arm. Baseline demographics, clinical characteristics, and prior ARV use were generally balanced in both treatment arms (see Additional file 1: Table S1). Prior to their ARV regimen at screening, 41% of all patients had used ≥ 1 other PI, 42% had used ≥ 1 other nucleos(t)ide reverse transcriptase inhibitor, and 30% had used ≥ 1 nonnucleoside reverse transcriptase inhibitor.

### Efficacy

**Fig. 1:**
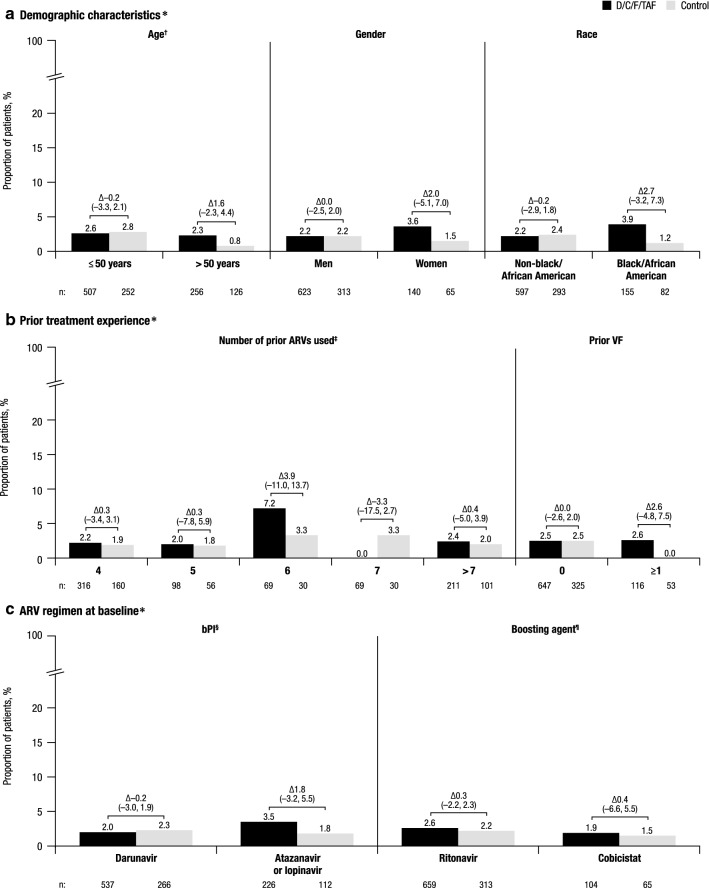
Virologic rebound rates through week 48 by subgroup. *D/C/F/TAF* darunavir/cobicistat/emtricitabine/tenofovir alafenamide, *ARV* antiretroviral, *VF* virologic failure, *bPI* boosted protease inhibitor, *CI* confidence interval. *Differences (95% CI) in virologic rebound rate between treatment arms are reported above the brackets. The total number of patients in each treatment arm for each subgroup is reported below the x-axis labels. ^†^For patients ≤ 50 years without polypharmacy, virologic rebound rates were 2.5% (11/436) with D/C/F/TAF and 2.9% (6/206) with control; for patients > 50 years with polypharmacy, these rates were 0% (0/108) with D/C/F/TAF and 2.0% (1/50) with control. ^‡^Data are not reported for the 1 patient who had used 3 prior ARVs. ^§^Darunavir with ritonavir or cobicistat, atazanavir with ritonavir or cobicistat, and lopinavir with ritonavir. ^¶^Ritonavir with darunavir, atazanavir, or lopinavir; and cobicistat with darunavir or atazanavir

**Fig. 2:**
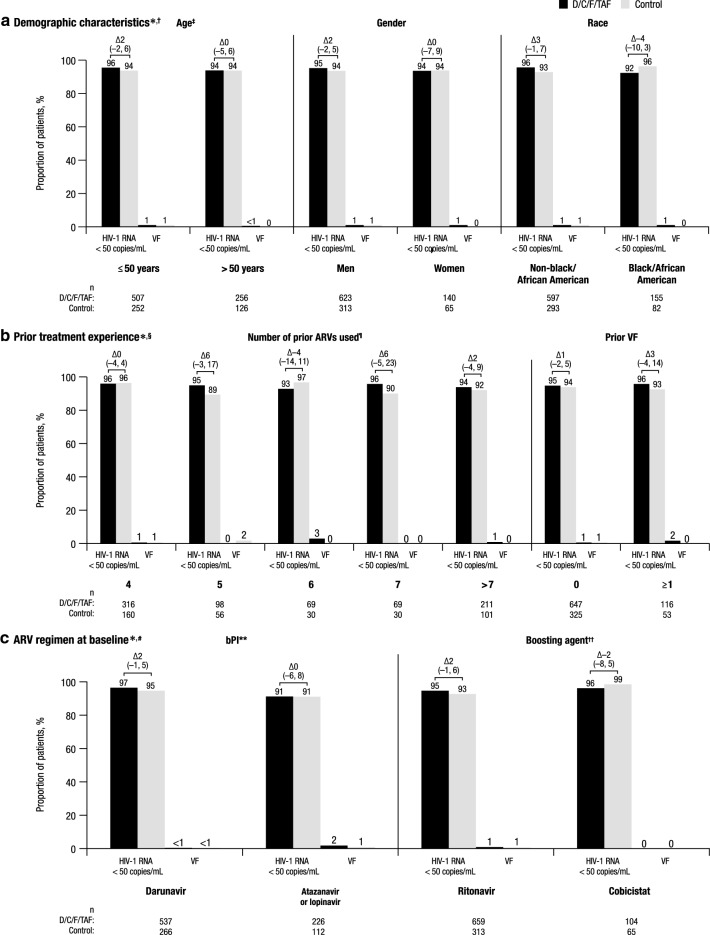
Virologic response and VF rates at week 48 by subgroup. *VF* virologic failure, *D/C/F/TAF* darunavir/cobicistat/emtricitabine/tenofovir alafenamide, *ARV* antiretroviral, *bPI* boosted protease inhibitor, *CI* confidence interval, *FDA* Food and Drug Administration. *Differences (95% CI) in virologic response rate between treatment arms are reported above the brackets. The total number of patients in each treatment arm for each subgroup is reported below the x-axis labels. ^†^For each subgroup, patients with missing virologic data (per FDA snapshot) in the D/C/F/TAF and control arms, respectively, were as follows: 4% and 6% for those ≤ 50 years, 6% and 6% for those > 50 years, 4% and 6% for men, 5% and 6% for women, 4% and 7% for those who are non-black/African American, and 7% and 4% for those who are black/African American. ^‡^For patients ≤ 50 years without polypharmacy, rates of HIV-1 RNA < 50 copies/mL were 96% (419/436) with D/C/F/TAF and 94% (193/206) with control; for patients > 50 years with polypharmacy, these rates were 93% (100/108) with D/C/F/TAF and 92% (46/50) with control. ^§^For each subgroup, patients with missing virologic data (per FDA snapshot) in the D/C/F/TAF and control arms, respectively, were as follows: 3.5% and 3.1% of those who used 4 prior ARVs, 5.1% and 8.9% of those who used 5 prior ARVs, 4.3% and 3.3% of those who used 6 prior ARVs, 4.3% and 10.0% of those who used 7 prior ARVs, 5.2% and 7.9% of those who used > 7 prior ARVs, 4.6% and 5.5% of those with 0 prior VFs, and 2.6% and 7.5% of those with ≥ 1 prior VF. ^¶^Data are not reported for the 1 patient who used 3 prior ARVs. ^#^For each subgroup, patients with missing virologic data (per FDA snapshot) in the D/C/F/TAF and control arms, respectively, were as follows: 3% and 5% for the darunavir group, 7% and 8% for the atazanavir or lopinavir group, 4% and 7% for the ritonavir group, 4% and 2% for the cobicistat group. **Darunavir with ritonavir or cobicistat, atazanavir with ritonavir or cobicistat, and lopinavir with ritonavir. ^††^Ritonavir with darunavir, atazanavir, or lopinavir; and cobicistat with darunavir or atazanavir

Cumulative virologic rebound rates were low in the D/C/F/TAF and control arms and similar across all subgroups evaluated, ranging from 0.0 to 7.2% with D/C/F/TAF and 0.0 to 3.3% with control (Fig. 1). Consistent with these results, virologic response rates were high in the D/C/F/TAF and control arms and similar across all subgroups, ranging from 91 to 97% with D/C/F/TAF and 89 to 99% with control (Fig. 2). Cumulative virologic rebound rates and virologic response rates for patients ≤ 50 years without polypharmacy and patients > 50 years with polypharmacy also fell within these ranges. Together, these study results suggest that the efficacy of D/C/F/TAF was not impacted by patients’ demographic characteristics, prior treatment experience, or ARV regimen at the time of treatment switch.

### Resistance

Four patients who rebounded had post-baseline genotypes available; 1 patient (D/C/F/TAF arm) had used 8 prior ARVs, 1 patient (control arm) had used 6 prior ARVs, and 2 patients (control arm) had used 4 prior ARVs. None of these 4 patients had a history of prior VF. No darunavir, primary PI, emtricitabine, or tenofovir RAMs were observed in these patients or any arm across subgroups [5].

### Safety

The incidence of AEs was similar in the D/C/F/TAF and control arms across all subgroups, except for a numerically higher incidence of study drug-related AEs with D/C/F/TAF relative to control (Table 1 and see Additional file 1: Tables S2 and S3). Rates of discontinuation due to AEs and rates of serious AEs were low with both D/C/F/TAF and control across subgroups, regardless of relatedness to study drug (in the overall population, 1% of patients in each arm discontinued due to AEs and 5% of patients in each arm had a serious AE [5]). The most common study drug-related AEs (≥ 2% in either arm) were diarrhea (D/C/F/TAF, 2%; control, 1%) and osteopenia (D/C/F/TAF, 1%; control, 2%) [5].

**Table 1:** Summary of AEs through week 48 by demographic characteristics

Parameter, n (%)	Age subgroups^a^				Gender subgroups				Race subgroups			
	≤ 50 years		> 50 years		Men		Women		Non-black/African American		Black/African American	
	D/C/F/TAF	Control	D/C/F/TAF	Control	D/C/F/TAF	Control	D/C/F/TAF	Control	D/C/F/TAF	Control	D/C/F/TAF	Control
n	507	252	256	126	623	313	140	65	597	293	155	82
Any AE	418 (82)	207 (82)	207 (81)	104 (83)	510 (82)	260 (83)	115 (82)	51 (79)	496 (83)	241 (82)	120 (77)	67 (82)
Related	103 (20)	15 (6)	35 (14)	13 (10)	110 (18)	26 (8)	28 (20)	2 (3)	112 (19)	21 (7)	23 (15)	7 (9)
Serious AEs	20 (4)	9 (4)	15 (6)	9 (7)	31 (5)	14 (5)	4 (3)	4 (6)	31 (5)	13 (4)	4 (3)	5 (6)
Related	0	0	1 (< 1)^b^	0	1 (< 1)	0	0	0	1 (< 1)	0	0	0
Grade 3–4 AEs	32 (6)	20 (8)	20 (8)	11 (9)	42 (7)	26 (8)	10 (7)	5 (8)	45 (8)	26 (9)	6 (4)	5 (6)
Related	7 (1)	1 (< 1)	3 (1)	3 (2)	8 (1)	4 (1)	2 (1)	0	8 (1)	4 (1)	2 (1)	0
AEs leading to discontinuation	6 (1)	1 (< 1)	5 (2)	4 (3)	10 (2)	5 (2)	1 (1)	0	8 (1)	5 (2)	3 (2)	0
Related	5 (1)	0	3 (1)^c^	3 (2)	7 (1)	3 (1)	1 (1)	0	7 (1)	3 (1)	1 (1)	0

*AE* adverse event, *D/C/F/TAF* darunavir/cobicistat/emtricitabine/tenofovir alafenamide

^a^Rates of related AEs in the D/C/F/TAF and control arms, respectively, for patients ≤ 50 years without polypharmacy were: 0% (0/436) and 0% (0/206) with serious related AEs; 1% (6/436) and 1% (1/206) with grade 3–4 related AEs; 1% (5/436) and 0% (0/206) discontinued due to related AEs. Rates of related AEs in the D/C/F/TAF and control arms, respectively, for patients > 50 years with polypharmacy were: 0% (0/108) and 0% (0/50) with serious related AEs; 0% (0/108) and 2% (1/50) with grade 3–4 related AEs; 0% (0/108) and 2% (1/50) discontinued due to related AEs

^b^This patient had grade 3 pancreatitis; this AE led to discontinuation

^c^Each patient had 1 study drug-related AE leading to discontinuation (pancreatitis, increase alanine aminotransferase, and urticaria)

**Fig. 3:**
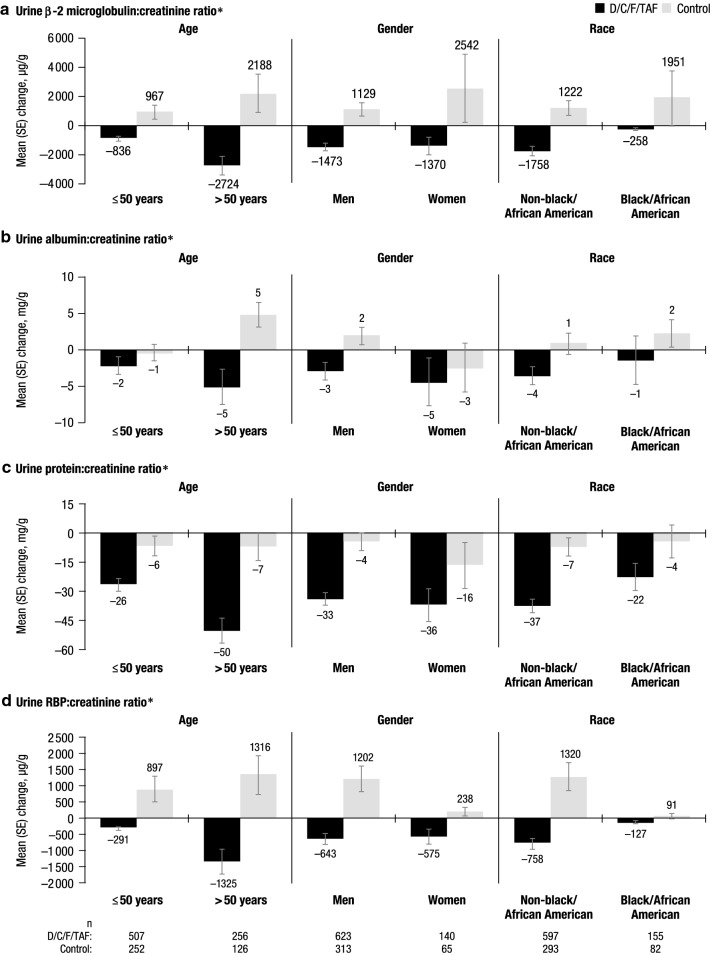
Changes from baseline to week 48 in renal laboratory parameters based on demographic characteristics. *D/C/F/TAF* darunavir/cobicistat/emtricitabine/tenofovir alafenamide, *SE* standard error, *RBP* retinol binding protein. *The total number of patients in each treatment arm for each subgroup is reported at the bottom of the figure

Across subgroups based on age, gender, and race, mean changes in markers of proteinuria from baseline to week 48 generally decreased in the D/C/F/TAF arm and increased in the control arm (Fig. 3). Over 48 weeks, estimated glomerular filtration rate (based on serum cystatin C) remained generally stable with D/C/F/TAF across these demographic subgroups (see Additional file 1: Figure S1). Median changes in lipid values from baseline to week 48 were also generally similar across demographic subgroups in the D/C/F/TAF arm, and across subgroups in the control arm (see Additional file 1: Figure S2).

**Fig. 4:**
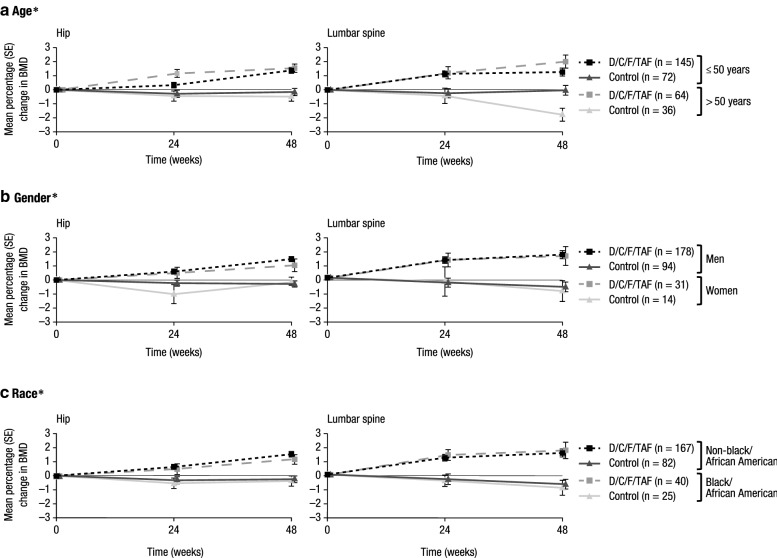
Changes from baseline in BMD over time (hip and lumbar spine) based on demographic characteristics. *BMD* bone mineral density, *D/C/F/TAF* darunavir/cobicistat/emtricitabine/tenofovir alafenamide, *SE* standard error. *Data are from the bone investigation substudy, which included 209 patients in the D/C/F/TAF arm and 108 patients in the control arm. The total number of substudy patients in each treatment arm for each subgroup is reported in the legends

In the bone investigation substudy, increases in BMD over time in the hip, lumbar spine, and femoral neck were observed with D/C/F/TAF across subgroups based on age, gender, and race (Fig. 4 and see Additional file 1: Figure S3). There were no fractures unrelated to trauma in any arm across subgroups [5].

## Discussion

EMERALD allowed treatment-experienced, virologically suppressed patients with varied treatment histories to enroll, and thus switching from a bPI + emtricitabine/TDF to D/C/F/TAF was evaluated in a population more reflective of the real world than other recent switch studies [6–8]. In the current analysis of EMERALD, low rates of virologic rebound and high virologic response rates were observed regardless of demographic characteristics, prior treatment experience (including prior VF), and ARV regimen at baseline in patients who switched to D/C/F/TAF compared with continuing on their baseline regimen. Efficacy results with D/C/F/TAF were generally similar to other recent switch studies [6–8], although direct comparisons are difficult due to differences in inclusion criteria and the resulting study populations.

No resistance to any D/C/F/TAF component was observed in EMERALD, which is consistent with the established high barrier to resistance of darunavir [2]. This result is important because among virologically suppressed patients, resistance mutations may be archived and thus have the potential to reemerge at the time of virologic rebound. Reemergent resistance is a particular concern among individuals with prior VF. In EMERALD, plasma samples were taken at baseline for potential future exploratory research, and genoarchive analysis of these samples has been performed for patients who rebounded and those with prior VF [10].

D/C/F/TAF was associated with a favorable safety and tolerability profile in the overall EMERALD population and across all subgroups. Improved renal function and bone safety were observed with D/C/F/TAF compared with control in the overall population over 48 weeks [5], consistent with the switch from TDF to TAF in this arm [11–13], and results were generally similar regardless of age, gender, or race. Furthermore, changes in lipid parameter values through week 48 did not vary by demographic characteristics and, as previously described [5], low proportions of patients initiated lipid-lowering therapy during the study, with no significant differences between treatment arms. The higher proportion of patients with ≥ 1 study drug-related AE among those who switched to D/C/F/TAF, compared with those who continued their current regimen in the control arm, has been observed in prior switch studies [14].

Accelerated aging has been associated with HIV-1 infection, and polypharmacy and aging-related comorbidities, such as osteopenia/osteoporosis and increased bone fracture risk, are clinical concerns [15–18]. Therefore, evaluation of safety parameters for different age groups is important in studies of new HIV-1 treatment regimens. In the current analysis of EMERALD, among patients > 50 years with polypharmacy in the D/C/F/TAF arm, none discontinued due to a related AE (and none experienced virologic rebound). Overall, the bone safety profile of D/C/F/TAF was generally consistent in patients ≤ 50 and > 50 years at week 48. Bone safety results with D/C/F/TAF were also consistent in women and men after 48 weeks, which was reassuring given that women may experience an increased risk of bone events, particularly after menopause [19, 20]. There were few fractures in the overall population and, more importantly, all reported fractures were trauma-related [5].

A limitation of this study was the smaller number of patients in some of the subgroups who are historically underrepresented in clinical trials (e.g., > 50 years, women, black/African American) [21]. Nevertheless, the overall findings from this analysis show that switching to D/C/F/TAF may be an option for a broad range of virologically suppressed patients with HIV-1, in particular those who are experienced with multiple ARV agents and/or have had prior VF, as well as those currently on multi-tablet regimens of lopinavir, atazanavir, or darunavir. Moreover, the consistent results regardless of bPI used at baseline, together with the advantages of darunavir compared with other PIs [3, 22], suggest that D/C/F/TAF, the only PI-based STR, is a viable treatment choice for virologically suppressed patients currently on a PI-based regimen.

## Conclusions

Switching to D/C/F/TAF may be an effective strategy for individuals who are stably suppressed and would like to simplify therapy, including patients with prior experience with multiple ARVs or a history of prior VF (without a history of darunavir RAMs or VF on a darunavir-based regimen).

## Additional file

**Additional file 1: Table S1** Baseline demographic and disease characteristics. **Table S2.** Summary of AEs week 48 by prior treatment experience. **Table S3.** Summary of AEs through week 48 by ARV regimen at baseline. **Figure S1.** Mean change in eGFR_cystC_ from baseline to week 48. **Figure S2.** Lipid values at baseline and week 48 by demographic subgroups. **Figure S3.** Changes from baseline in femoral neck BMD over time based on demographic characteristics.

## Data Availability

The data sharing policy of Janssen Pharmaceutical Companies of Johnson & Johnson is available at https://www.janssen.com/clinical-trials/transparency. As noted on this site, requests for access to the study data can be submitted through Yale Open Data Access (YODA) Project site at http://yoda.yale.edu.

## Abbreviations

AE: adverse event
ARV: antiretroviral
BMD: bone mineral density
bPI: boosted protease inhibitor
CI: confidence interval
D/C/F/TAF: darunavir/cobicistat/emtricitabine/tenofovir alafenamide
FDA: US Food and Drug Administration
HIV: human immunodeficiency virus
QD: once-daily
RAM: resistance-associated mutation
STR: single-tablet regimen
TDF: tenofovir disoproxil fumarate
VF: virologic failure
VL: viral load
