# Supplementary material for: Darunavir/cobicistat/emtricitabine/tenofovir alafenamide in treatment-experienced, virologically suppressed patients with HIV-1: subgroup analyses of the phase 3 EMERALD study
Source: AIDS Res Ther. 2019 Aug 29;16:23. doi: 10.1186/s12981-019-0235-1 (PMC6716878; doi:10.1186/s12981-019-0235-1)
Supplement: Supplementary file 1 — Additional file 1: Table S1 Baseline demographic and disease characteristics. Table S2. Summary of AEs week 48 by prior treatment experience. Table S3. Summary of AEs through week 48 by ARV regimen at baseline. Figure S1. Mean change in eGFRcystC from baseline to week 48. Figure S2. Lipid values at baseline and week 48 by demographic subgroups. Figure S3. Changes from baseline in femoral neck BMD over time based on demographic characteristics. [file 12981_2019_235_MOESM1_ESM.docx]

**Additional File**

**File Format: Word document**

**Description: Additional tables and figures**

Table S1. Baseline demographic and disease characteristics.^*^

|  | **D/C/F/TAF**  **n = 763** | **Control**  **n = 378** |
| --- | --- | --- |
| **Demographic characteristics** |  |  |
| Age, median (range), y | 46 (19–75) | 45 (20–78) |
| Age category, n (%) |  |  |
| ≤50 y | 507 (66) | 252 (67) |
| >50 y | 256 (34) | 126 (33) |
| Gender, n (%) |  |  |
| Men | 623 (82) | 313 (83) |
| Women | 140 (18) | 65 (17) |
| Race, n (%)^†^ |  |  |
| Non-black/African American | 597 (79) | 293 (78) |
| Black/African American | 155 (21) | 82 (22) |
| **Disease characteristics** |  |  |
| CD4^+^ cell count, median (range), cells/μL | 630 (111–1,921) | 624 (131–1,764) |
| Number of prior ARVs used, n (%)^‡^ |  |  |
| 4^§^ | 316 (41) | 160 (42) |
| 5 | 98 (13) | 56 (15) |
| 6 | 69 (9) | 30 (8) |
| 7 | 69 (9) | 30 (8) |
| >7 | 211 (28) | 101 (27) |
| Prior VF, n (%) |  |  |
| 0 | 647 (85) | 325 (86) |
| ≥1 | 116 (15) | 53 (14) |
| Time since diagnosis, median (range), y | 9.34 (0.6–35.0) | 8.94 (0.6–32.6) |
| Patients with 4 prior ARVs used^§^ | 4.46 (0.6–29.8) | 4.35 (0.6–27.7) |
| Patients with >7 prior ARVs used | 19.75 (3.9–33.9) | 18.96 (3.7–31.6) |
| Patients with 0 prior VFs | 7.78 (0.6–33.9) | 7.48 (0.6–32.6) |
| Patients with ≥1 prior VF | 17.96 (3.6–35.0) | 18.12 (1.8–31.0) |
| Time since first ARV therapy, median (range), y | 6.23 (0.6–32.9) | 5.75 (0.6–27.5)^¶^ |
| Patients with 4 prior ARVs used^§^ | 3.51 (0.6–24.6) | 3.49 (0.6–15.9) |
| Patients with >7 prior ARVs used | 17.28 (3.2–32.9) | 16.51 (2.9–27.5) |
| Patients with 0 prior VFs | 5.20 (0.6–32.9) | 4.66 (0.6–26.7) |
| Patients with ≥1 prior VF | 16.00 (3.3–24.4) | 14.78 (1.7–27.5) |
| bPI used at baseline, n (%)^#^ |  |  |
| Darunavir | 537 (70) | 266 (70) |
| Boosted by ritonavir | 439 (58) | 202 (53) |
| Boosted by cobicistat | 98 (13) | 64 (17) |
| Atazanavir | 167 (22) | 82 (22) |
| Boosted by ritonavir | 161 (21) | 81 (21) |
| Boosted by cobicistat | 6 (1) | 1 (<1) |
| Lopinavir^**^ | 59 (8) | 30 (8) |

ARV, antiretroviral; VF, virologic failure; bPI, boosted protease inhibitor.

^*^Overall, 275 (24%) patients had polypharmacy (D/C/F/TAF, n = 179; control, n = 96).

^†^Percentages calculated excluding patients with “unknown” or “not reported” race.

^‡^Includes ARVs used at screening and boosting agents. One (<1%) patient was included in the study, despite having only 3 prior ARVs used, due to a data recording error; this patient was in the control arm and was excluded from the subgroup analyses.

^§^Four prior ARVs is the ARV regimen used at screening (ie, bPI + emtricitabine/tenofovir disoproxil fumarate).

^¶^n = 377.

^#^Percentages may not total 100% due to rounding.

^**^Boosted by ritonavir.

Table S2. Summary of AEs Week 48 by prior treatment experience.

| **Parameter, n (%)** | **Number of prior ARVs used^*^** | | | | | | | | | | **Prior VF** | | | |
| --- | --- | --- | --- | --- | --- | --- | --- | --- | --- | --- | --- | --- | --- | --- |
|  | **4** | | **5** | | **6** | | **7** | | **>7** | | **0** | | **≥1** | |
|  | **D/C/F/TAF** | **Control** | **D/C/F/TAF** | **Control** | **D/C/F/TAF** | **Control** | **D/C/F/TAF** | **Control** | **D/C/F/TAF** | **Control** | **D/C/F/TAF** | **Control** | **D/C/F/TAF** | **Control** |
| n | 316 | 160 | 98 | 56 | 69 | 30 | 69 | 30 | 211 | 101 | 647 | 325 | 116 | 53 |
| Any AE | 270  (85) | 135  (84) | 80  (82) | 40  (71) | 50  (73) | 28  (93) | 51  (74) | 24  (80) | 174  (83) | 84  (83) | 530  (82) | 267  (82) | 95  (82) | 44  (83) |
| Related | 56  (18) | 10  (6) | 19  (19) | 3  (5) | 12  (17) | 4  (13) | 11  (16) | 0 | 40  (19) | 11  (11) | 110  (17) | 24  (7) | 28  (24) | 4  (8) |
| Serious AEs | 15  (5) | 6  (4) | 2  (2) | 4  (7) | 0 | 2  (7) | 3  (4) | 2  (7) | 15  (7) | 4  (4) | 28  (4) | 14  (4) | 7  (6) | 4  (8) |
| Related | 0 | 0 | 0 | 0 | 0 | 0 | 0 | 0 | 1  (<1) | 0 | 0 | 0 | 1  (1) | 0 |
| Grade 3–4 AEs | 25  (8) | 14  (9) | 2  (2) | 4  (7) | 3  (4) | 3  (10) | 4  (6) | 3  (10) | 18  (9) | 7  (7) | 45  (7) | 24  (7) | 7  (6) | 7  (13) |
| Related | 7  (2) | 0 | 0 | 1  (2) | 0 | 0 | 0 | 0 | 3  (1) | 3  (3) | 8  (1) | 2  (1) | 2  (2) | 2  (4) |
| AEs leading to discontinuation | 4  (1) | 1  (1) | 1  (1) | 1  (2) | 1  (1) | 0 | 0 | 1  (3) | 5  (2) | 2  (2) | 9  (1) | 3  (1) | 2  (2) | 2  (4) |
| Related | 3  (1) | 0 | 1  (1) | 1  (2) | 1  (1) | 0 | 0 | 0 | 3  (1) | 2  (2) | 6  (1) | 1  (<1) | 2  (2) | 2  (4) |

AE, adverse event; ARV, antiretroviral; VF, virologic failure; D/C/F/TAF, darunavir/cobicistat/emtricitabine/tenofovir alafenamide.

^*^Data are not reported for the 1 patient who used 3 prior ARVs.

Table S3. Summary of AEs through Week 48 by ARV regimen at baseline.

| **Parameter, n (%)** | **bPI at baseline^*^** | | | | **Boosting agent at baseline^†^** | | | |
| --- | --- | --- | --- | --- | --- | --- | --- | --- |
|  | **Darunavir** | | **Atazanavir or lopinavir** | | **Ritonavir** | | **Cobicistat** | |
|  | **D/C/F/TAF** | **Control** | **D/C/F/TAF** | **Control** | **D/C/F/TAF** | **Control** | **D/C/F/TAF** | **Control** |
| n | 537 | 266 | 226 | 112 | 659 | 313 | 104 | 65 |
| Any AE | 449 (84) | 218 (82) | 176 (78) | 93 (83) | 542 (82) | 259 (83) | 83 (80) | 52 (80) |
| Related | 93 (17) | 18 (7) | 45 (20) | 10 (9) | 128 (19) | 24 (8) | 10 (10) | 4 (6) |
| Serious AEs | 19 (4) | 13 (5) | 16 (7) | 5 (5) | 29 (4) | 16 (5) | 6 (6) | 2 (3) |
| Related | 1 (<1) | 0 | 0 | 0 | 1 (<1) | 0 | 0 | 0 |
| Grade 3–4 AEs | 33 (6) | 19 (7) | 19 (8) | 12 (11) | 47 (7) | 25 (8) | 5 (5) | 6 (9) |
| Related | 8 (1) | 1 (<1) | 2 (1) | 3 (3) | 10 (2) | 4 (1) | 0 | 0 |
| AEs leading to discontinuation | 4 (1) | 2 (1) | 7 (3) | 3 (3) | 10 (2) | 5 (2) | 1 (1) | 0 |
| Related | 3 (1) | 0 | 5 (2) | 3 (3) | 7 (1) | 3 (1) | 1 (1) | 0 |

AE, adverse event; ARV, antiretroviral; bPI, boosted protease inhibitor; D/C/F/TAF, darunavir/cobicistat/emtricitabine/tenofovir alafenamide.

^*^Darunavir with ritonavir or cobicistat, atazanavir with ritonavir or cobicistat, and lopinavir with ritonavir.

**^†^**Ritonavir with darunavir, atazanavir, or lopinavir; and cobicistat with darunavir or atazanavir.

Figure S1. Mean change in eGFR_cystC_ from baseline to Week 48.^*^


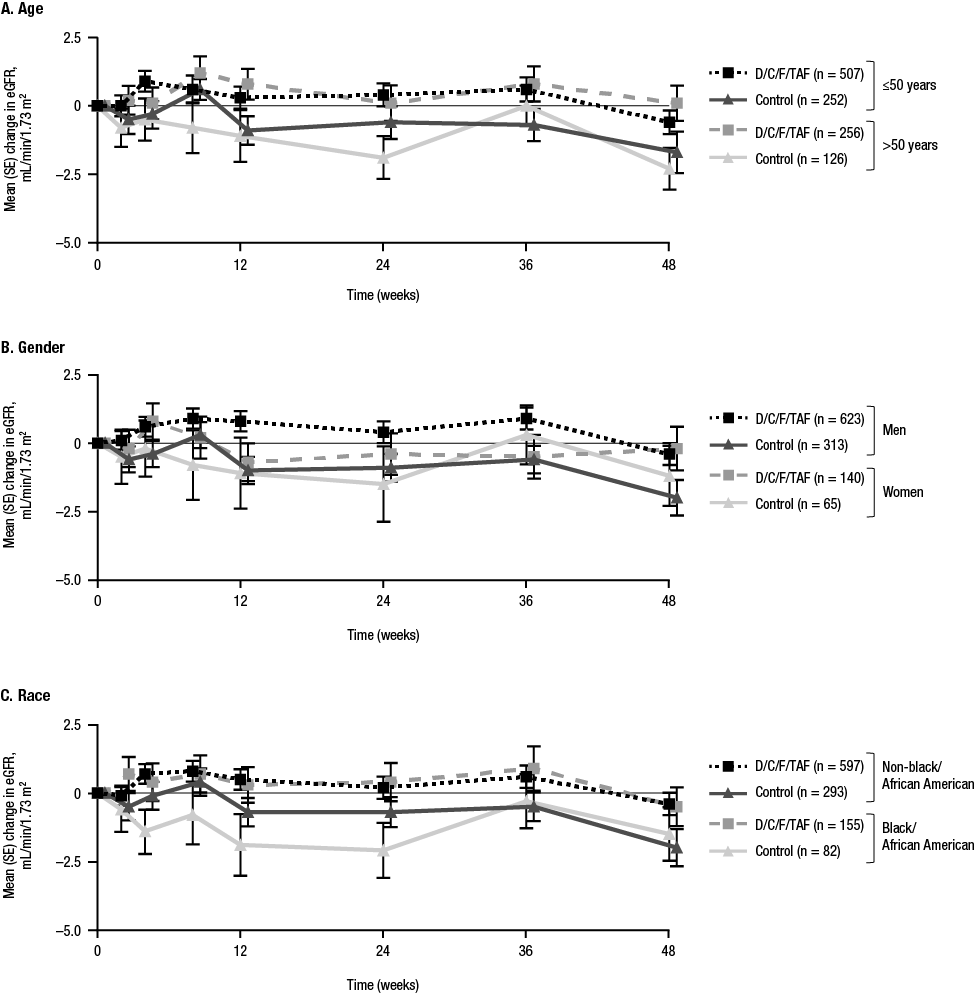


GFR_cystC_, estimated glomerular filtration rate based on serum cystatin C; SE, standard error; D/C/F/TAF, darunavir/cobicistat/emtricitabine/tenofovir alafenamide.

^*^eGFR_cystC_ by Chronic Kidney Disease Epidemiology Collaboration. The total number of patients in each treatment arm for each subgroup is reported in the legends.

Figure S2. Lipid values at baseline and Week 48 by demographic subgroups.^*^


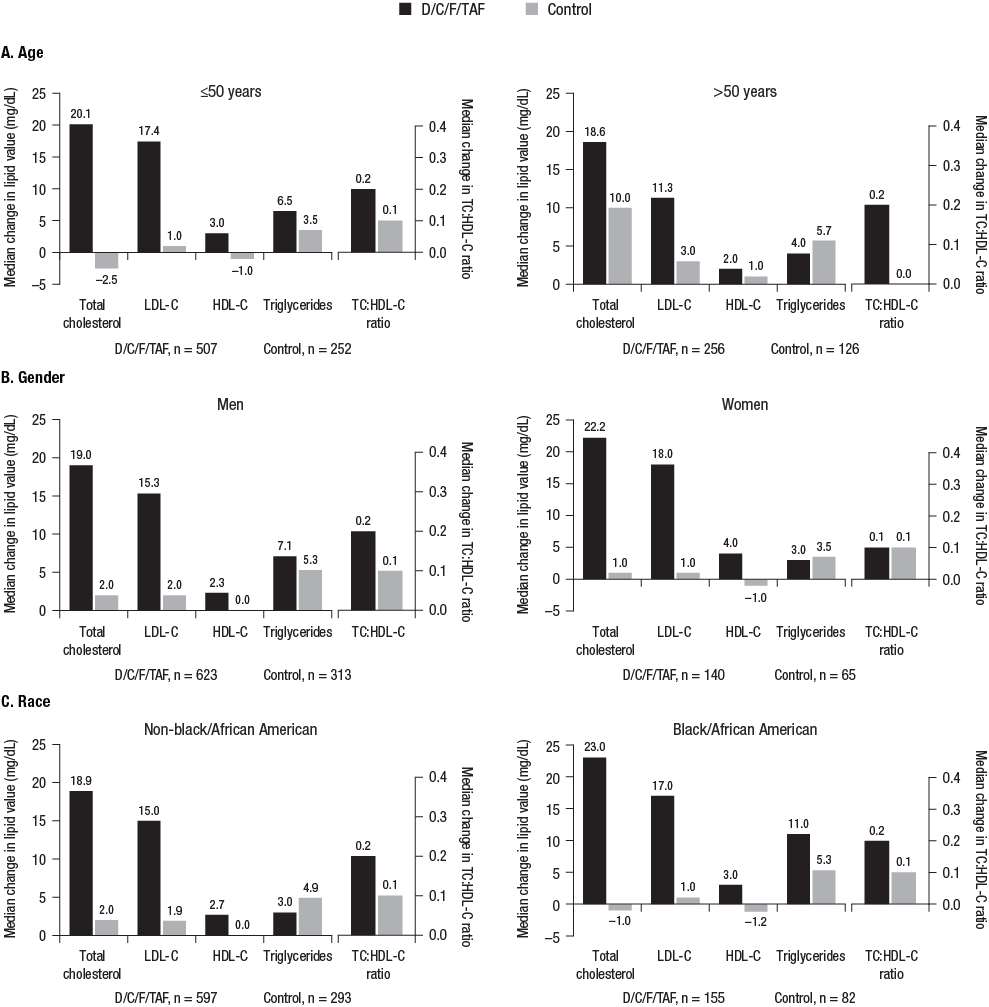


D/C/F/TAF, darunavir/cobicistat/emtricitabine/tenofovir alafenamide; LDL-C, low-density lipoprotein cholesterol; HDL-C, high-density lipoprotein cholesterol; TC, total cholesterol.

^*^The total number of patients in each treatment arm for each subgroup is reported below the x-axis labels.

Figure S3. Changes from baseline in femoral neck BMD over time based on demographic characteristics.^*^


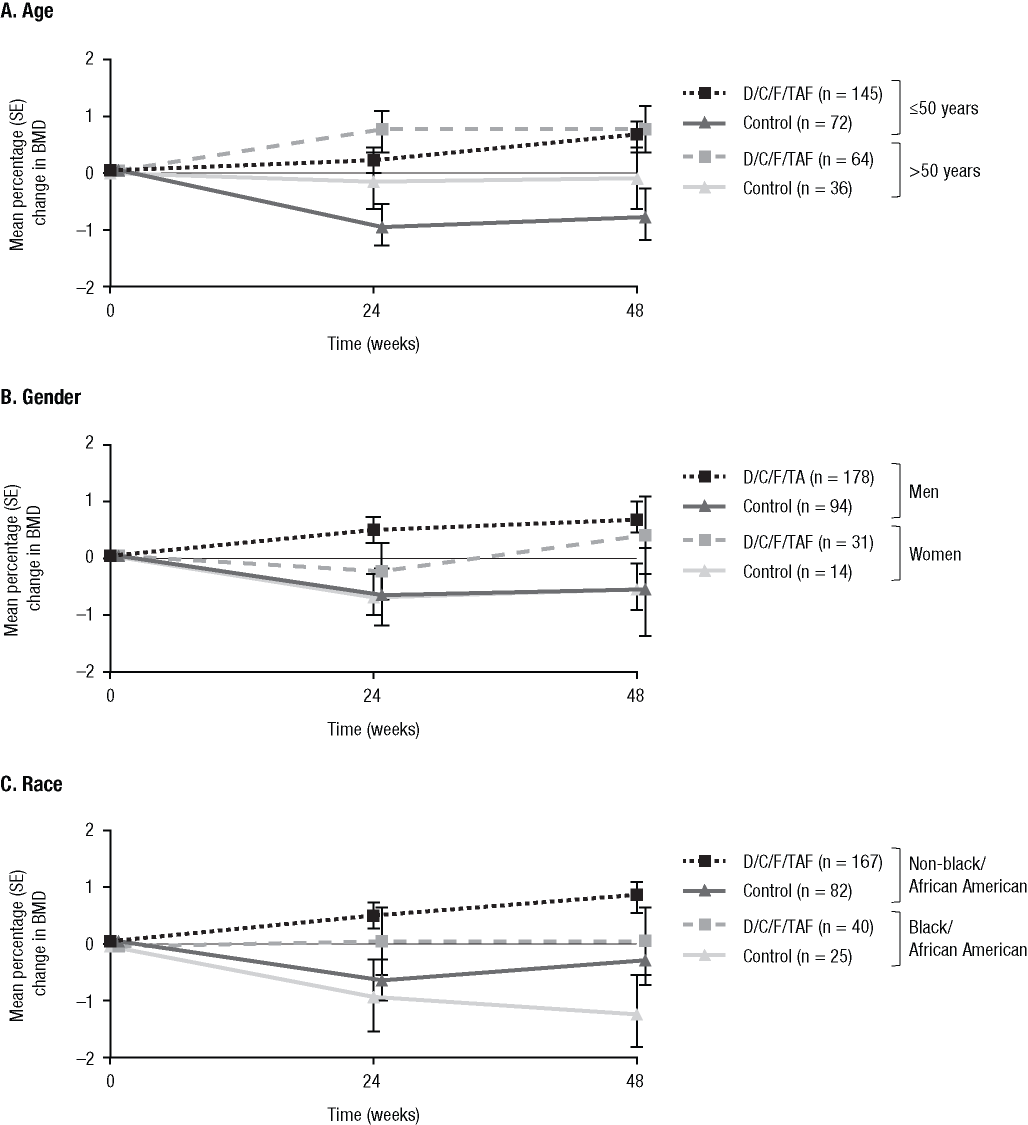


BMD, bone mineral density; D/C/F/TAF, darunavir/cobicistat/emtricitabine/tenofovir alafenamide; SE, standard error.

^*^Data are from the bone investigation substudy, which included 209 patients in the D/C/F/TAF arm and 108 patients in the control arm. The total number of substudy patients in each treatment arm for each subgroup is reported in the legends.
